# Influences of surface treatments with abrasive paper and sand-blasting on surface morphology, hydrophilicity, mineralization and osteoblasts behaviors of n-CS/PK composite

**DOI:** 10.1038/s41598-017-00571-4

**Published:** 2017-04-03

**Authors:** Xiaoming Tang, Kai Huang, Jian Dai, Zhaoying Wu, Liang Cai, Lili Yang, Jie Wei, Hailang Sun

**Affiliations:** 1 Department of Orthopedics, Huai’an First People’s Hospital, Nanjing medical university, Huai’an, 223001 Jiangsu Province PR China; 2 Department of orthopedics, Shanghai Zhabei Central Hospital, Shanghai, 200070 PR China; 3 Key Laboratory for Ultrafine Materials of Ministry of Education, East China University of Science and Technology, Shanghai, 200237 PR China; 4 Department of Orthopedic Surgery, Changzheng Hospital, Second Military Medical University, Shanghai, 20003 PR China

## Abstract

The surfaces of nano-calcium silicate (n-CS)/polyetheretherketone (PK) composites were treated with abrasive paper and sand-blasting, and the surfaces performances of the as-treated composites were studied. The results showed that the surface roughness, hydrophilicity and mineralization of the simulated body fluid (SBF) of the composites surfaces were significantly improved, and the properties of the composites treated by with sand-blasting were better than those treated with abrasive paper. Moreover, the treated composites significantly promoted osteoblasts responses, such as cell attachment, spreading, proliferation and alkaline phosphatase (ALP) activity, compared to un-treated composites, and the cellular responses to the composites treated with sand-blasting were better than those treated with abrasive paper. The results suggested that surface treatment with sand-blasting was an effective method to greatly improve the surface bioperformances of the n-CS/PK composite, and this treated composite with improved bioactivity and cytocompatibility might be a promising implant material for orthopedic applications.

## Introduction

Since receiving US Food and Drug Administration (FDA) approval in the late 1990s, polyetheretherketone (PK) has been extensively used as an implantable material for spinal, trauma, and orthopedic applications due to its good mechanical properties, chemical resistance, high thermal stability and biocompatibility, etc^1, 2^. Moreover, PK implants were found to have biomechanical properties (especially elastic modulus) that resemble the natural bone of human beings, which could effectively reduce stress shielding that is often observed in conventional metallic implants, such as biomedical titanium alloys, cobalt-bases alloys and stainless steel^3, 4^. However, PK implants are comprised of bioinert materials that cannot bond to bone tissue (osseointegration) after they are implanted *in vivo*, and an encapsulation of fibrous tissue will form that isolates implants from the surrounding bone, which would cause loosening/abscission of the implants from host bone and ultimate failure of the implants^5, 6^.

Osseointegration can ensure that the implants bond with natural bone through biochemical reactions at the interface between the materials and the bone, which favors the implants fixation in host bone^7^. During the past few years, to enhance the osseointegration performance of PK, bioactive coatings, such as plasma spraying and plasma ion deposition, have been applied to modify PK to create bioactive surfaces for implants with improved biological properties^8–10^. However, the fragility and low mechanical strength as well as the weak bonding to the PK substrates of the coating have caused unsatisfactory osseointegration between the implants and bone tissue. Moreover, various bioactive materials, such as bioglass, hydroxyapatite, titania and calcium silicate, have been incorporated into PK to develop bioactive composites for bone implants^11, 12^.

In previous studies, we prepared several PK-based bioactive composites, including hydroxyapatite, titania and calcium silicate, and we investigated their chemical-physical-biological properties^11–14^. It was found that although incorporation of bioactive materials into PK improved the mechanical performance and bioactivity of the composites, most of the bioactive materials were dispersed into the PK matrix, and the composite surface was significantly covered by PK (bioactive materials were not exposed on the composite surfaces). Thus, the surface bioactivities of these composites were lower than bioactive materials (such as calcium silicate, bioglass, hydroxyapatite, etc.) alone. Surface treatment offers an effective way to enhance surface biological properties while preserving the advantages of the materials. Therefore, proper surface treatments should be presented as alternative solutions. In this study, a nano-calcium silicate (n-CS)/polyetheretherketone (PK) composite was fabricated and surface treated using abrasive paper and sand-blasting. The effects of surface treatment of bone implants comprised of n-C/PK composite on surface morphology/microstructure, hydrophilicity, mineralization and osteoblast behaviors were evaluated.

## Results

### Surface morphology and roughness of composites after treatment

**Figure 1:**
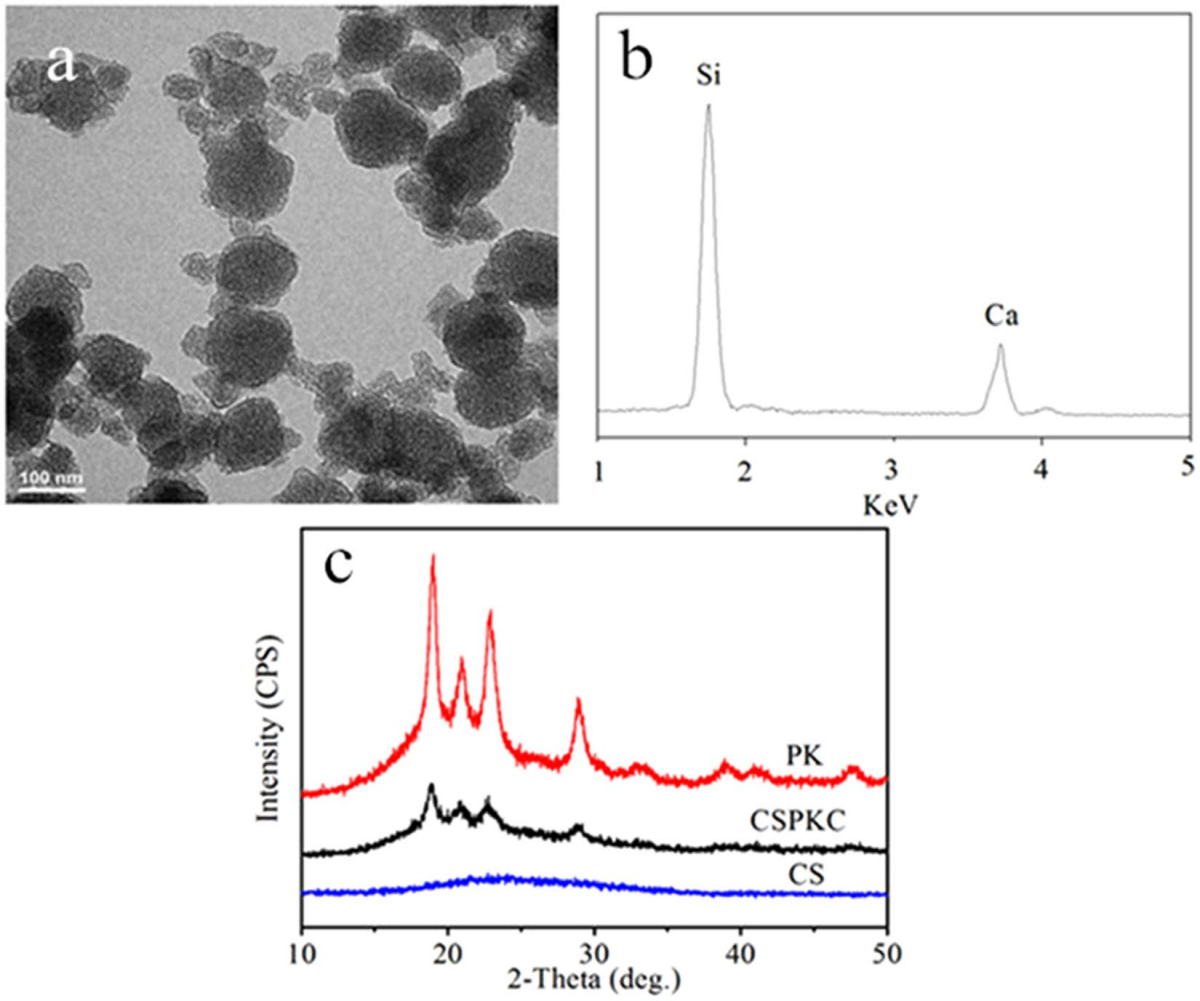
TEM image (**a**) and EDS (**b**) of nano calcium silicate (n-CS), and XRD (**c**) of PK, n-CS/PK composite and n-CS.

Figure 1 shows the TEM image (a) and EDS (b) of nano-calcium silicate (n-CS), and the XRD (c) of PK, n-CS/PK composite and n-CS. The globular n-CS particles were approximately 100 nm, and contained Ca and Si elements. In addition, the peaks at 18.71°, 20.76°, 22.81° and 28.83° belonged to PK. After n-CS particles were added into PK, the positions of the peaks did not change, but the intensity of the peaks of PK in the composites significantly reduced, which indicated that the crystallinity of PK decreased after incorporation of n-CS.

**Figure 2:**
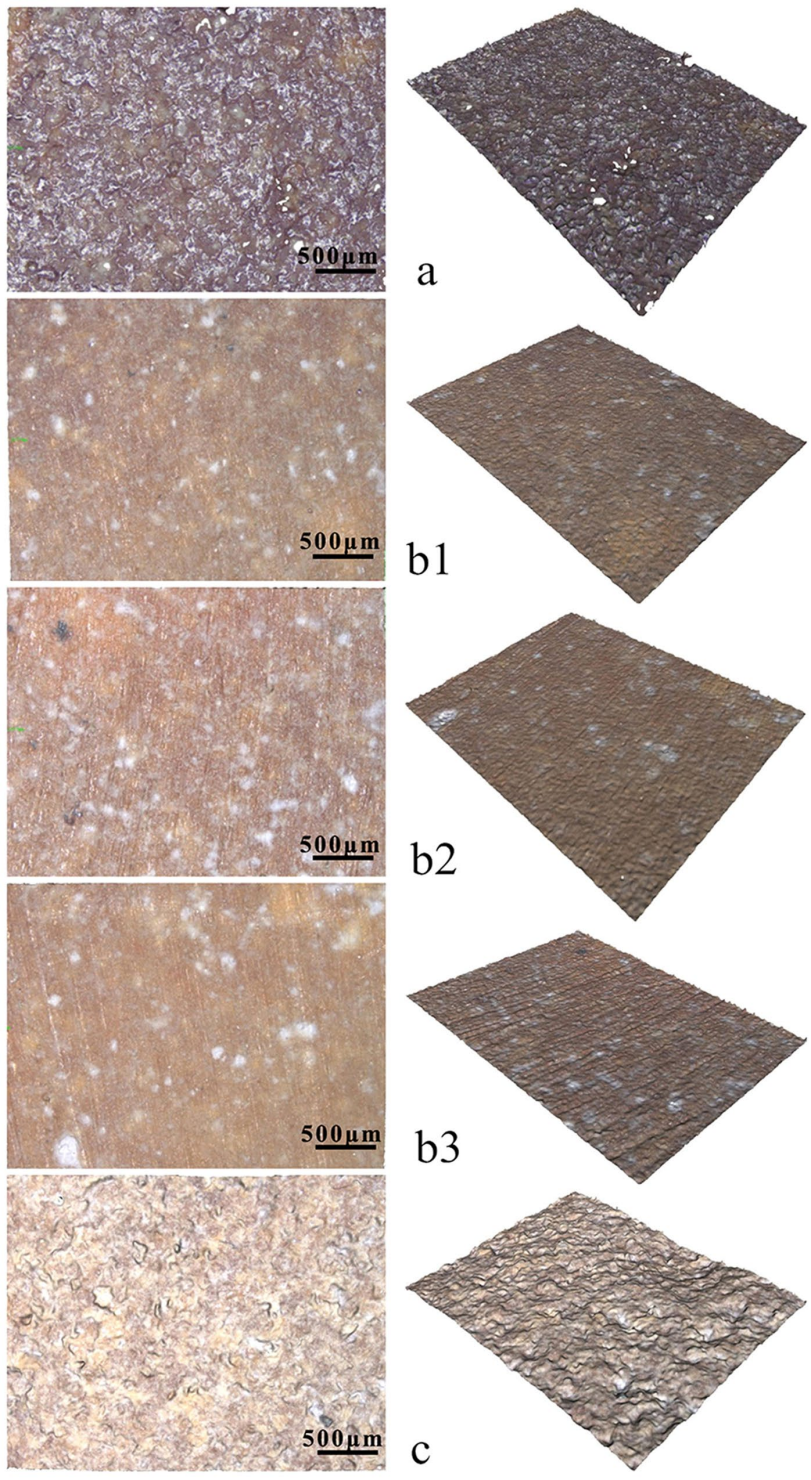
The 2D (left) and 3D (right) optical images of CSPKC (**a**), 12-CSPKC (**b1**), 8-CSPKC (**b2**), 4-CSPKC (**b3**) and sb-CSPKC (**c**).

Figure 2 shows the 2D and 3D optical images of the surface morphology/microstructure of CSPKC, 12-CSPKC, 8-CSPKC, 4-CSPKC and sb-CSPKC determined by InfiniteFocus Optical 3D surface metrology, in which the n-CS/PK composite is CSPKC, and abrasive paper treatments 1200#, 800# and 400# mesh are labeled as 12-CSPKC, 8-CSPKC and 4-CSPKC, and treatment with sand-blasting is sb-CSPKC. CSPKC exhibited a rough surface, and some PK materials covered the composite surface before treatment. However, the composites of 12-CSPKC, 8-CSPKC and 4-CSPKC showed smooth surfaces after treatment with abrasive paper. Furthermore, the sb-CSPKC treatment produced a rough surface after treatment with sand-blasting, which was different from CSPKC. Table S1 shows the surface roughness of the composites before and after treatment with abrasive paper or sand-blasting. It was found that the Ra values for the surface roughness of CSPKC, 12-CSPKC, 8-CSPKC, 4-CSPKC and sb-CSPKC were 1.58 μm, 1.06 μm, 1.13 μm, 1.48 μm and 3.82 μm, respectively.

**Figure 3:**
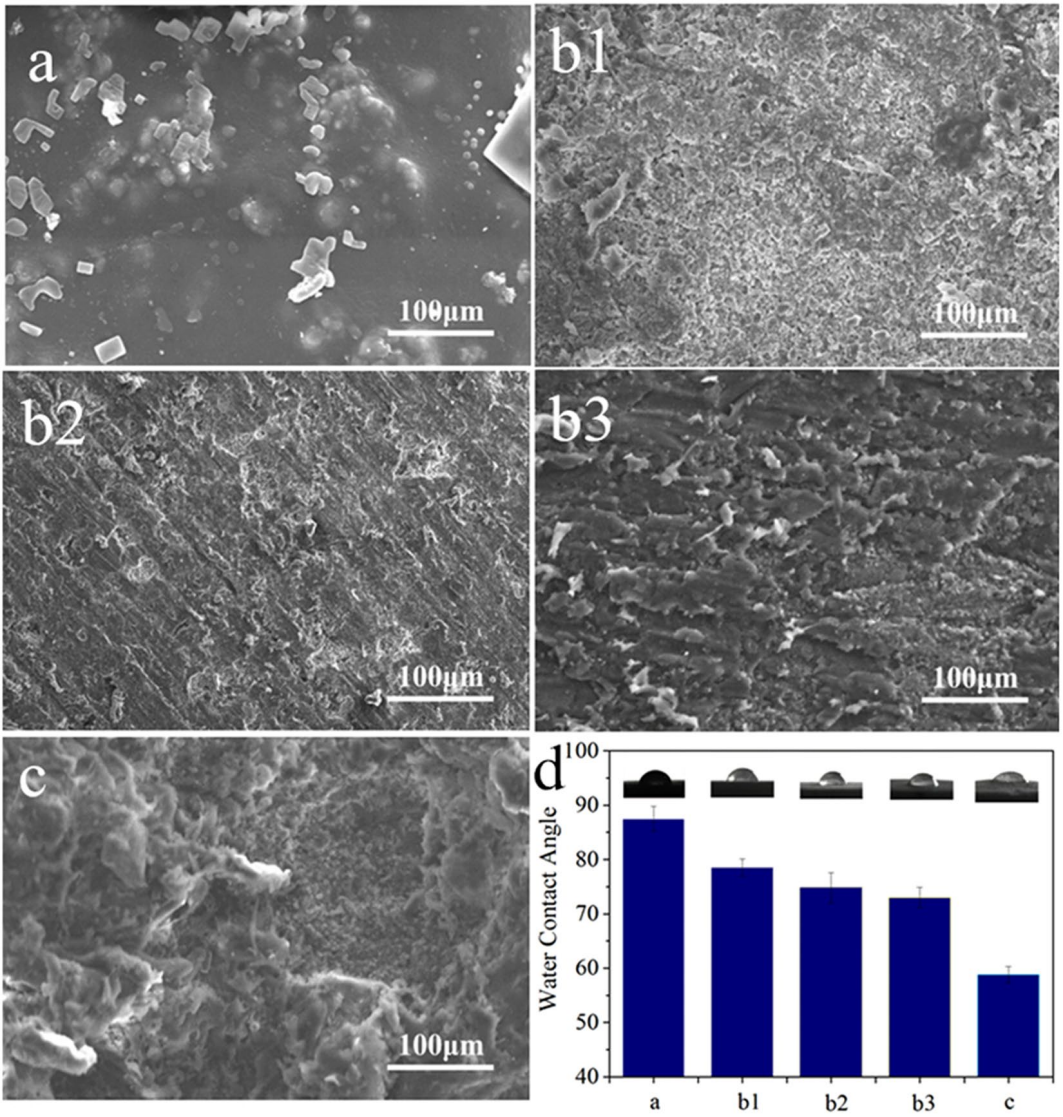
SEM images of surface morphology/microstructure and water contact angles (**d**) of CSPKC (**a**), 12-CSPKC (**b1**), 8-CSPKC (**b2**), 4-CSPKC (**b3**) and sb-CSPKC (**c**).

Figure 3 presents SEM images of the surface morphology/microstructure of the composites before and after treatment with abrasive paper or sand-blasting. PK covered on the CSPKC surface, which had a coarse surface, and only a few n-CS particles were exposed on the composite surface before treatment. However, 12-CSPKC, 8-CSPKC and 4-CSPKC showed smooth surfaces. Additionally, many n-CS particles were exposed on the surfaces after treatment with abrasive paper, many pores (from several μm to approximately 100 μm) appeared on the sb-CSPKC surface, and a large number of n-CS particles were exposed on the surfaces after treatment with sand-blasting.

### Hydrophilic properties of composites after treatment

Figure 3d shows the water contact angles of the composites before and after treatment with abrasive paper or sand-blasting. The water contact angles of CSPKC, 12-CSPKC, 8-CSPKC, 4-CSPKC and sb-CSPKC were 87.5°, 78.5°, 74.8°, 73° and 58.8°, respectively. Compared to CSPKC, the water contact angles of the composites surfaces significantly decreased after treatment with abrasive paper (12-CSPKC < 8-CSPKC < 4-CSPKC, decreased with meshes) or sand-blasting, and the water contact angles of sb-CSPKC were the lowest among all the samples.

### Mineralization of composites in SBF

**Figure 4:**
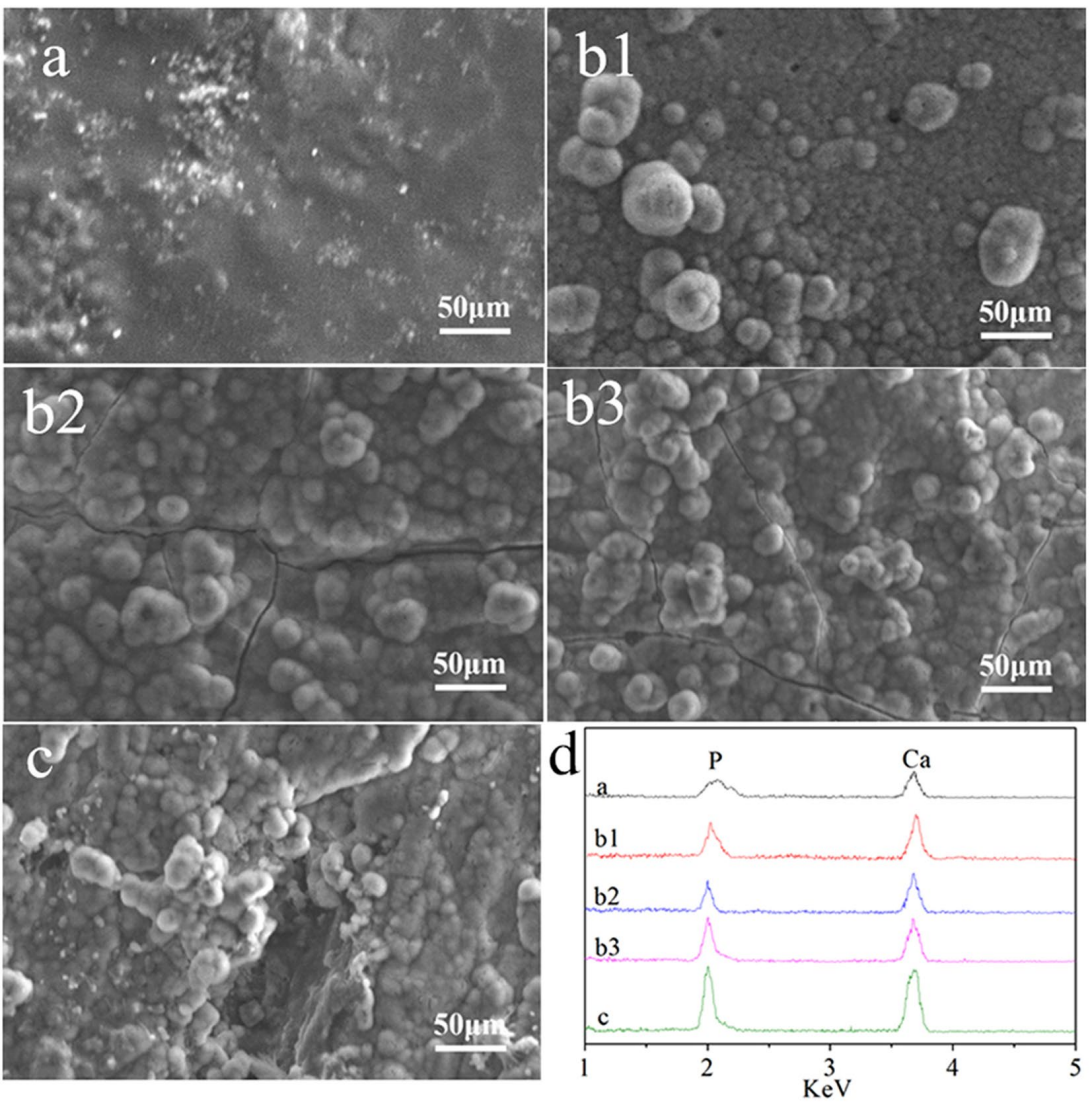
SEM images of surface morphology and EDS of surface elements (**d**) of CSPKC (**a**), 12-CSPKC (**b1**), 8-CSPKC (**b2**), 4-CSPKC (**b3**) and sb-CSPKC (**c**) after soaking in SBF for 7 days.

Figure 4 shows the SEM images of the surface morphology/microstructures of the composites treated with abrasive paper or sand-blasting after immersion in SBF for 7 days. Many globular particles appeared on the surfaces of 12-CSPKC, 8-CSPKC, 4-CSPKC and sb-CSPK, which were typical apatite particles, and the apatite layer covered the composites surfaces. However, only small numbers of apatite particles were found on the CSPKC surface.

Figure 4d shows the EDS of the composites surfaces treated with abrasive paper or sand-blasting after immersion in SBF for 7 days. The Ca and P peaks appeared on the surfaces of CSPKC, 12-CSPKC, 8-CSPKC, 4-CSPKC and sb-CSPK.

**Figure 5:**
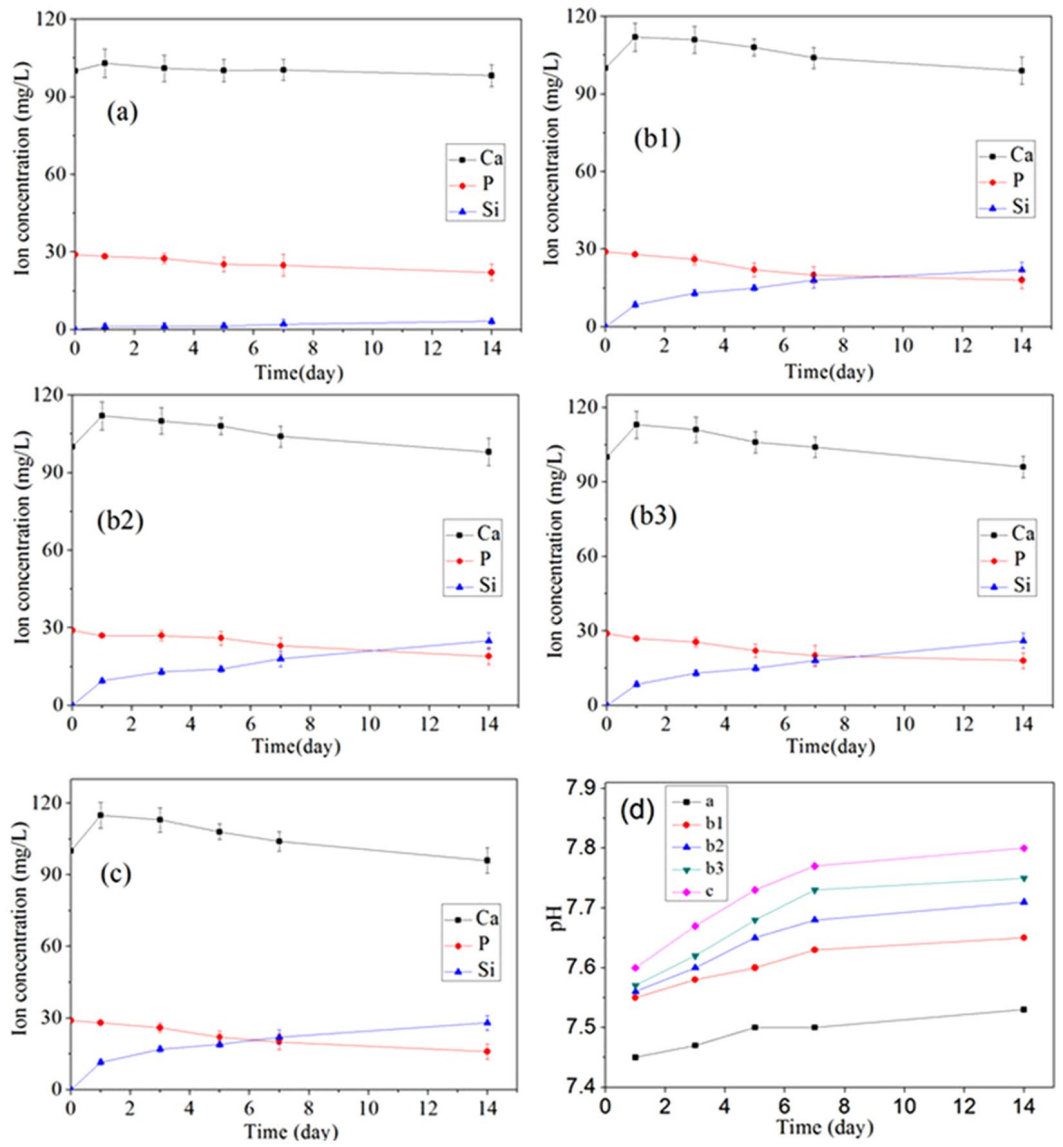
Changes in ion concentrations in solution and pH changes of the solution (**d**) after CSPKC (**a**), 12-CSPKC (**b1**), 8-CSPKC (**b2**), 4-CSPKC (**b3**) and sb-CSPKC (**c**) were immersed in SBF for different periods of time.

Figure 5 shows the changes in ion concentrations of Ca, Si and P in solutions after the composites soaked in SBF for different times. The ion concentrations of Ca for 12-CSPKC, 8-CSPKC, 4-CSPKC and sb-CSPK increased on first day, and then gradually decreased over 14 days. In addition, the P ions gradually decreased while the Si ions gradually increased for 12-CSPKC, 8-CSPKC, 4-CSPKC and sb-CSPK during the soaking time. However, for CSPKC, the ion concentrations of Ca and P decreased while Si increased, which showed there were small changes during the soaking time.

Figure 5d shows the pH variation of SBF solution containing CSPKC, 12-CSPKC, 8-CSPKC, 4-CSPKC and sb-CSPK for different periods of time. The pH of the solution containing CSPKC (7.5) showed no obvious changes during the soaking time. However, the pH of the solution containing 12-CSPKC, 8-CSPKC and 4-CSPKC slightly increased to 7.64, 7.70 and 7.75, while the pH of the solution for sb-CSPKC sharply increased to 7.8 after soaking for 14 days.

### Cell attachment

**Figure 6:**
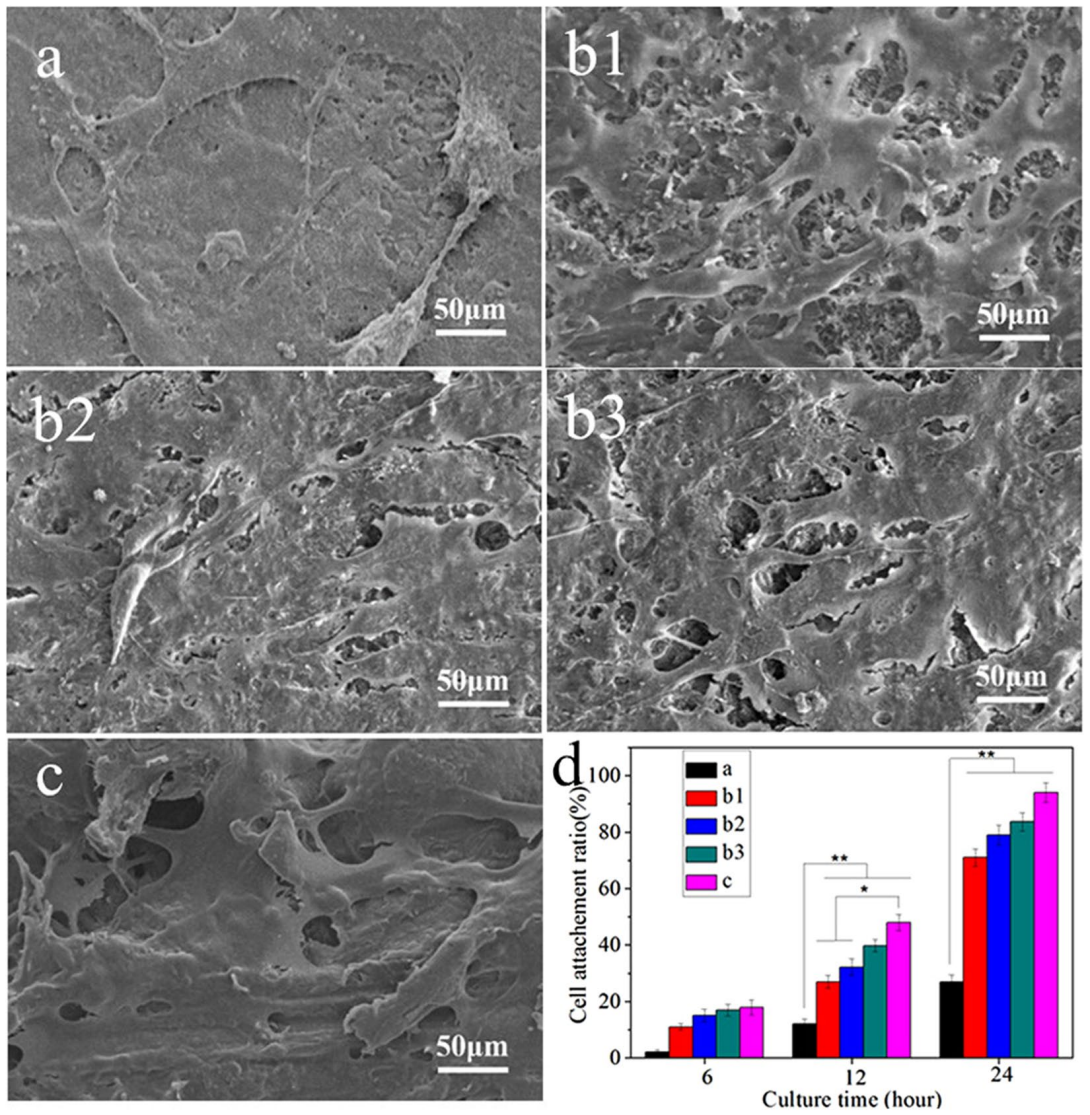
SEM images and cell attachment ratios (**d**) of MC3T3-E1 cells cultivated on the CSPKC (**a**), 12-CSPKC (**b1**), 8-CSPKC (**b2**), 4-CSPKC (**b3**) and sb-CSPKC (**c**) for 24 hours.

Figure 6d presents the attachment of the MC3T3-E1 cells on the composite surfaces before and after treatment with abrasive paper and sand-blasting. It was found that the attachments of the cells to all the samples increased with time, which indicated the composites had good cytocompatiblity. In addition, sb-CSPKC had the most cell attachment, while CSPKC had the lowest cell attachment at 6, 12, and 24 h, and the attachments of the cells to CSPKC treated with abrasive paper increased according to the mesh number (12-CSPKC <8-CSPKC <4-CSPKC).

Figure 6 shows the SEM images of MC3T3-E1 cells attached to specimens for 24 hours. A greater amount of cells grew on the composites (12-CSPKC, 8-CSPKC, 4-CSPKC and sb-CSPKC) with more efficient spreading, and many fine filopodia of the cells were anchored in the composites surfaces, whereas the cells on CSPKC were spindle shaped with few filopodia.

### Cell proliferation and cytoskeleton morphology

**Figure 7:**
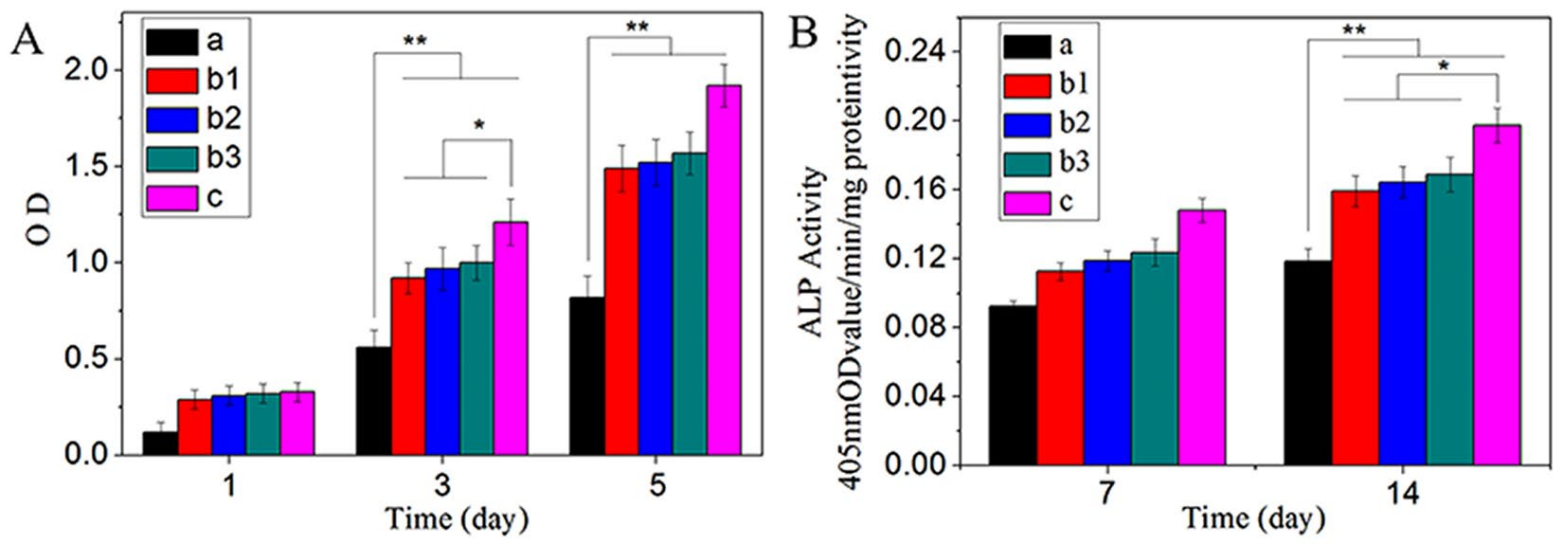
Proliferation (**A**) and ALP activity (**B**) of MC3T3-E1 cells cultivated on the CSPKC (**a**), 12-CSPKC (**b1**), 8-CSPKC (**b2**), 4-CSPKC (**b3**) and sb-CSPKC (**c**) for different times.

Figure 7A shows the proliferation of MC3T3-E1 cells cultured on the composites surface, which was analyzed using a CCK-8 assay. The proliferation of the cells on all the samples increased with time, which indicated the composites had good cytocompatiblity. In addition, the proliferation of the cells on the composites (12-CSPKC, 8-CSPKC, 4-CSPKC and sb-CSPKC) was significantly higher compared to CSPKC at 1, 3, and 5 days, and no significant differences were found between the composites treated with abrasive paper. Moreover, at 3 and 5 days, the proliferation of the cells on the composites (sb-CSPKC) treated with sand-blasting was higher compared to the composite treated with abrasive paper.

**Figure 8:**
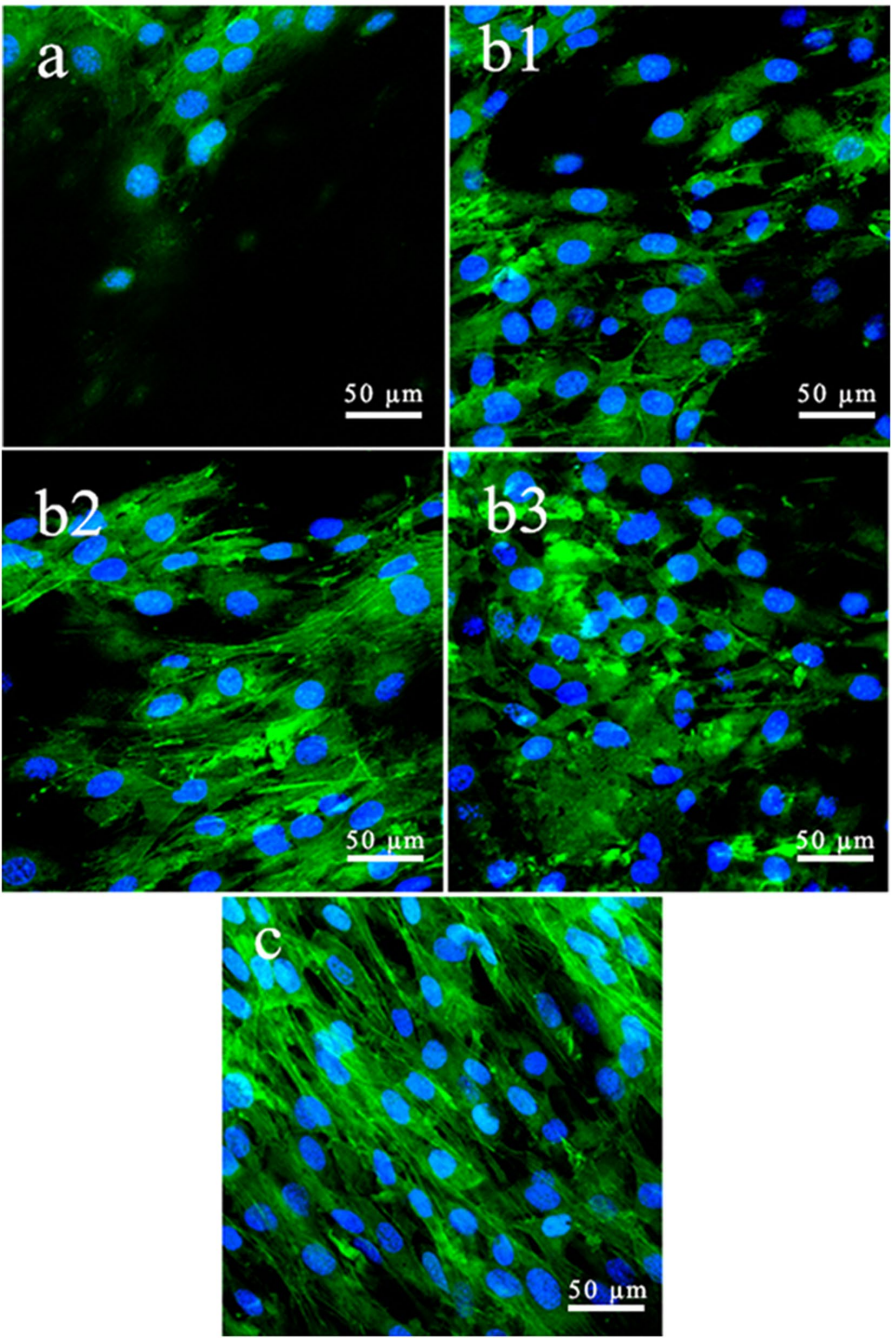
Confocal images of MC3T3-E1 cells cultivated on the specimens for 5 days.

Figure 8 presents CLSM images of the cytoskeleton of phalloidin-stained MC3T3-E1 cells on the composites surfaces. Many of the cells that spread and regularly grew on the composites (12-CSPKC, 8-CSPKC, 4-CSPKC and sb-CSPKC) were polygonal and clustered, whereas small numbers of cells grew irregularly on the CSPKC. Furthermore, more actin filaments linking adjacent cells were observed for cells that grew on the composites (12-CSPKC, 8-CSPKC, 4-CSPKC and sb-CSPKC) than on CSPKC.

### ALP activity assay

Figure 7B shows the ALP activities of the MC3T3-E1 cells cultured on composites surfaces at 7 and 14 days. The ALP activities of the cells on all the samples increased with time, which indicated the composites had good cytocompatiblity. In addition, the ALP activities of the cells on the composites (12-CSPKC, 8-CSPKC, 4-CSPKC and sb-CSPKC) treated with abrasive paper and sand-blasting were the higher than CSPKC at 7 and 14 days, and no significant differences were found for the composite treated with abrasive paper. Moreover, at 7 and 14 days, the ALP activity of the cells on sb-CSPKC was higher than the composites (12-CSPKC, 8-CSPKC and 4-CSPKC) treated with abrasive paper.

## Discussions

Polyetheretherketone (PK) has attracted increasing attention as an alternative material for hard tissue repair due to its good biocompatibility, mechanical properties and chemical stability, and it is currently used in orthopedic clinics^15^. However, PK is a bioinert material that does not promote cell adhesion and growth, and PK implants can barely bind to host bone directly, which impedes the use of PK in clinical treatments^16^.

Consequently, in this study, calcium silicate was incorporated into PK to develop n-CS/PK biocomposites (CSPKC) to enhance the bioactivity of PK, and the surfaces of the composites were treated with abrasive paper or sand-blasting. The results indicated that only a few n-CS particles were exposed on the CSPKC surface, which was covered by PK. However, after treatment with abrasive paper, the composites showed smooth and flattened surfaces, and many n-CS particles were exposed on the surfaces (most PK on the surfaces was removed). Thus, the surface roughness of Ra values of the composites decreased (12-CSPKC: 1.06 μm, CSPKC: 1.13 μm and 4-CSPKC: 1.48 μm) compared to CSPKC (1.58 μm). Clearly, the meshes of the abrasive paper had some effects on the surface roughness of the composites (the surface roughness of the composites increased with the decrease in meshes of the abrasive paper), but there were no obvious differences.

Moreover, a large number of n-CS particles were exposed on sb-CSPK surfaces after the PK was removed by sand-blasting and many pores (from several μm to approximately 100 μm) were made on the surfaces. Thus, the Ra of the surface roughness for sb-CSPK (3.82 μm) significantly increased compared to CSPKC, 12-CSPKC, 8-CSPKC and 4-CSPKC. The results suggested that sand-blasting was good way to treat composite surfaces because it made coarse surfaces with many micropores and several n-CS particles were exposed on the surfaces.

Surface hydrophilicity can greatly affect the bioperformances of biomaterial implants^17^. In this study, The water contact angles of the composites surfaces significantly decreased after treatment with abrasive paper and sand-blasting as compared to CSPKC (87.5°). The water contact angles of sb-CSPK were the lowest (58.8°) compared to 12-CSPKC (78.5°), 8-CSPKC (74.8°) and 4-CSPKC (73°).

The results showed that the surface treatment with abrasive paper or sand-blasting significantly improved the hydrophilicity of the composite surfaces, and sand-blasting proved to be a viable way to improve the hydrophilicity of the composite. It is well-known that PK is a hydrophobic polymeric material, while n-CS is a hydrophilic inorganic material. The improved hydrophilicity was likely due to the n-CS particles exposed on the composite surfaces after treatment. It has been reported that biomaterials surfaces are exposed to dilute serum and that more hydrophilic surfaces are better for cell attachment, spreading and proliferation than hydrophobic surfaces^18^. Therefore, the n-CS/PK composites treated with abrasive paper or sand-blasting likely had good hydrophilicity that had positive effects on the cells responses.

Bioactive materials, such as bioglass, hydroxyapatite (HA) and glass-ceramic A-W, have the ability to bond with bone tissues through the formation of an apatite-layer on surfaces *in vivo*, and the bone-bonding ability of these biomaterials is often evaluated by examining apatite mineralization on the surfaces in SBF *in vitro* ^19–21^. In this study, after the composites were immersed in SBF for 7 days, the apatite layers with many globular apatite particles covered the 12-CSPKC, 8-CSPKC, 4-CSPKC and sb-CSPKC surfaces. However, only small numbers of apatite particles were found on the CSPKC. In addition, the EDS demonstrated that Ca and P peaks appeared on the surfaces of CSPKC, 12-CSPKC, 8-CSPKC and 4-CSPKC and sb-CSPK, which indicated that the deposits were Ca-P substances.

The ion concentrations of Ca for 12-CSPKC, 8-CSPKC, 4-CSPKC and sb-CSPK increased on the first day, and then gradually decreased over 14 days after the composites were soaked in SBF, which indicated that the n-CS on the composite surfaces first dissolved and released Ca ions into SBF, and then the Ca-P substances were deposited. The formation of apatite will consume P ions. Thus, the P ions gradually decreased. In addition, the Si ions for 12-CSPKC, 8-CSPKC, 4-CSPKC and sb-CSPK gradually increased during the soaking time because of the dissolution of n-CS into the SBF. However, for CSPKC, small changes of Ca, P and Si ion concentrations in SBF were found during soaking time because only a small amount of these ions were released from n-CS dissolution into the SBF (only small CS particles were exposed on the CSPKC surface, which was covered by PK). Thus, a small amount of apatite was deposited on CSPKC.

The mechanism of apatite formation on the composite surfaces could be suggested as follows. After soaking into SBF, the composite initially released Ca^2+^, which was exchanged with H^+^ in solution and formed silanol (Si-OH) groups^22^. The Si-OH groups were negatively charged, which attracted Ca^2+^ by electrostatic interaction^23^. With Ca^2+^ accumulated on the composites surfaces, the PO_4_^3−^ in solution was attracted by Ca^2+^ and deposited on the surfaces, which triggered the formation of an apatite nucleus on the surfaces. The apatite nucleus continued to grow by consuming the Ca and P ions in the surrounding fluid to form an apatite layer^24^. The results indicated that surface treatment of abrasive paper and sand-blasting had significantly effects on the mineralization of composites *in vitro*. Abundant CS particles were exposed on the composite surfaces after treatment, which was conducive to the formation of an apatite layer.

The initial cell attachment and spreading will influence the proliferation and differentiation of cells on implants surfaces^25^. The surface roughness and hydrophilicity of biomaterials had effects on cell responses, and rough surfaces and the hydrophilic surfaces of biomaterials were better for cell attachment and spreading than smooth surfaces and hydrophobic surfaces^26^. In this study, a greater amount of MC3T3-E1 cells grew on 12-CSPKC, 8-CSPKC, 4-CSPKC and sb-CSPK with more efficient spreading, and many fine filopodia of the cells were anchored in the composite surfaces, whereas the cells for CSPKC were spindle-shaped with a few filopodia. Furthermore, the sb-CSPK with the highest surface roughness and hydrophilicity had the highest cell attachment, while CSPKC with the lowest surface roughness and hydrophilicity had the lowest cell attachment. The results demonstrated that sb-CSPK with rough surfaces and hydrophilic surfaces treated with sand-blasting obviously promoted cell attachment and spreading.

The proliferations of MC3T3-E1 cells on 12-CSPKC, 8-CSPKC, 4-CSPKC and sb-CSPK was significantly higher compared to CSPKC, and the proliferation on sb-CSPK was significantly higher than 12-CSPKC, 8-CSPKC and 4-CSPKC. Moreover, more actin filaments linking adjacent cells grew on 12-CSPKC, 8-CSPKC, 4-CSPKC and sb-CSPK, which were polygonal and clustered, whereas small numbers of cells were found to grow on CSPKC. It is known that ALP activity is a key marker of osteogenic differentiation of osteoblasts in the early stages. Higher levels of ALP activity secretion illustrated more rapid and earlier differentiation of cells cultured on the materials, and osteogenic differentiation of the cells around the interface between bone and implants played a crucial role in the new bone formation process. In this study, the ALP activities of MC3T3-E1 cells on 12-CSPKC, 8-CSPKC, 4-CSPKC and sb-CSPK were significantly higher than CSPKC, and the ALP activity of cells on sb-CSPK was significantly higher than 12-CSPKC, 8-CSPKC and 4-CSPKC. The results demonstrated that the sb-CSPK treated with sand-blasting effectively promoted the proliferation and differentiation of MC3T3-E1 cells.

Studies have shown that the formation of the apatite layer on the biomaterial surface was beneficial to osteoblast proliferation and facilitated osteoblasts differentiation to form an extracellular matrix composed of biological apatite and collagen^27, 28^. Moreover, appropriate concentrations of Si and Ca ions released from Ca-Si based biomaterials played important roles in stimulating the proliferation and differentiation of osteoblasts^29^. In this study, the results revealed that the apatite layers appeared on the composite surface, and Si and Ca ions were released from the composite after treatment with abrasive paper and sand-blasting, which promoted the osteoblast proliferation and differentiation. Moreover, the pH of the solution containing 12-CSPKC, 8-CSPKC and 4-CSPKC slightly increased to 7.64, 7.70 and 7.75, while the pH of the solution for sb-CSPKC sharply increased to 7.8 due to the dissolution of n-CS and the formation of a weak alkaline environment. Previous studies have shown that weak alkaline conditions would provide a better micro-environment for cell proliferation and subsequently differentiation^30^.

Therefore, it could be suggested that the mechanism of the positive response of MC3T3-E1 cells to the composites after treatment could be attributed to the improved surface roughness, hydrophilicity, apatite layer formation, Ca and Si ion release, and weak alkaline micro-environment. In short, an n-CS/PK composite treated with abrasive paper and sand-blasting could greatly improve bioperformance, which would facilitate the attachment, proliferation and differentiation of osteoblasts. The n-CS/PK biocomposite treated with sand-blasting might be a promising implanted material for orthopedic applications.

## Conclusions

In this study, bone implants made of n-CS/PK composites were surface treated using abrasive paper and sand-blasting. The results showed that surface treatments (n-CS was exposed on the composite surfaces) obviously decreased the water contact angles while greatly improving the mineralization ability of the composites in SBF compared to un-treated composites. In addition, the surface roughness of the composite treated by sand-blasting was obviously enhanced compared to abrasive paper. Moreover, the composites treated with abrasive paper and sand-blasting significantly promoted cell attachment, spreading and proliferation, and improved the ALP activity of MC3T3-E1 cells compared to un-treated composite; moreover, the sand-blasting treatment was better than the abrasive paper. With improved surface roughness, hydrophilicity, bioactivity and cytocompatibility, the n-CS/PK biocomposite treated with sand-blasting might be a promising implanted material for orthopedic applications.

## Methods

### Preparation of n-CS and n-CS/PK composite

First, 4 g of P123 (EO20PO70EO20, 5800, Sigma Aldrich) was dissolved by stirring in a H_2_O (30 mL) and HCl solution (120 mL, 2.0 M, Shanghai Lingfeng Chemical Reagent Co., Ltd.) in a water bath for 1 hour. Then, magnesium nitrate hexahydrate (9.6 g) was added to the solution, and tetraethyl orthosilicate (TEOS, 9.12 mL, Shanghai Lingfeng Chemical Reagent Co., Ltd.) was added dropwise to the solution with magnetical stirring at 50 °C for 5 hours. The obtained white suspension was placed under a fume hood at room temperature for 24 hours for precipitation, which was then isolated by centrifugation, washed thoroughly with deionized water, and dried at 60 °C. The obtained powders were calcinated in air at 600 °C for 6 hours at a heating rate of 1 °C/min to obtain nano-magnesium silicate (n-MS). PK powders were obtained from Victrex Manufacturing Ltd. (South Yorkshire, UK). The n-CS/PK composite with 40 w% n-CS was produced (the mixture of n-CS and PK powders) using an injection-molding machine (BA-300/050CD, Battenfeld, Awans, Belgium) with an injection temperature of 380 °C.

### Surface treatment of composites

The n-CS/PK composite was cut into discs (Φ10 × 2 mm), and the sample surfaces were treated using abrasive paper or sand-blasting. The n-CS/PK composite was CSPKC, the abrasive paper treatments with 1200#, 800# and 400# mesh were called 12-CSPKC, 8-CSPKC and 4-CSPKC, respectively, and treatment with sand-blasting was called sb-CSPKC. The surface-treated composite samples were cleaned with deionized water for 2 hours in an ultrasonic oscillator (B3500S-MT, Branson, Danbury, Connecticut, USA). After drying at 37 °C overnight, the samples were sterilized with ethylene oxide for 3 hours, and the sterilized samples were sealed in sterile containers for subsequent use.

The hydrophilic properties of the surfaces of n-CS/PK composite samples before and after treatment with abrasive paper or sand-blasting were determined by measuring the water contact angles using the sessile drop method with a drop-shape analysis system (JC-2000D3, Shanghai Zhongcheng Digital Technology Co., Shanghai, China). The surface morphology/microstructure and chemical composition of n-CS/PK composite samples before and after treatment were characterized using scanning electron microscopy (SEM; S-4800, Hitachi, Tokyo, Japan) and energy dispersive spectrometry (EDS; X-Max, Horiba, Kyoto, Japan). The surface morphology/microstructure and surface roughness of Ra (arithmetical mean deviation of the profile) were determined with InfiniteFocus Optical 3D surface metrology (Inifinite Focus G4, Alicona, Austria).

### Mineralization of composites in SBF

The *in vitro* mineralization of the composite samples before and after treatment with abrasive paper or sand-blasting in simulated body fluid (SBF) was evaluated by examining the apatite formation at different times^31^. The samples (Φ10 × 2 mm), including CSPKC (a), 12-CSPKC (b1), 8-CSPKC (b2), 4-CSPKC (b3) and sb-CSPKC (c), were immersed into SBF (pH = 7.4) at 37 °C for 1, 3, 5, 7 and 14 days. At the end of each time point, the samples were taken out and rinsed 3 times with deionized water and dried overnight. Then, the surfaces of the composites were analyzed with SEM and EDS. Moreover, the changes in ion concentrations of Ca, P and Si in the solution after the samples were soaked in SBF for different times were tested by ICP-AES (IRIS 1000, Thermo Elemental, USA). The pH variation of the solution after the samples were soaked in SBF during the entire period was monitored by using a pH meter (FE20, Mettler Toledo).

### Cell attachment

The MC3T3-E1 cells (a mouse pre-osteoblastic cell line derived from mouse calvaria) were cultured in Dulbecco’s Modified Eagle’s Medium (DMEM; Hyclone, Thermo Fisher Scientific Inc., Miami, Florida, USA) supplemented with 10% fetal bovine serum (FBS; GibcoBRL, Grand Island, New York, USA), 1% penicillin (100 U/mL; GibcoBRL) and streptomycin sulfate (100 mg/mL; GibcoBRL) at 37 °C in a humidified atmosphere with 5% CO_2_ and 95% air. The culture medium was changed every 3 days.

The cell counting kit-8 (CCK-8) assay was used to analyze cell attachment to the samples at 6, 12 and 24 hours. The cultured MC3T3-E1 cells were digested with 0.25% trypsin (Sigma-Aldrich, Saint Louis, Missouri, USA), resuspended and seeded into a 24-well plate at a density of 3 × 10^4^/cm^2^ with three empty wells containing 1 mL of DMEM as blank controls. At each time point, the samples were gently rinsed three times with phosphate-buffered saline (PBS, pH = 7.4) to remove unattached cells and transferred to a new 24-well plate. A total of 50 μL of CCK-8 solution (Dojindo Molecular Technologies Inc., Kumamoto, Japan) was added to each well and incubated for 3 h. After that incubation, 100 μL of the supernatant was transferred into a 96-well plate and read at 450 nm using a microplate reader (Synergy HT, Biotek, Winooski, VT, USA). The mean absorbance value/optical density (OD) obtained from the blank control was subtracted from the ODs of the test groups.

After culturing on the samples for 24 hours, the MC3T3-E1 cells were fixed with 2.5% glutaraldehyde for 15 min and washed 3 times with PBS. The samples were dehydrated with an ethanol gradient at volume fractions of 30%, 50%, 70%, 90% and 100%, which were then replaced by hexamethyldisilazane (HMDS, Sigma-Aldrich). Finally, the samples were air-dried and observed with SEM.

### Cell proliferation and morphology

Cell proliferation was investigated using the CCK-8 assay. The seeding density of the cells was 1 × 10^4^/cm^2^, and the detection time points were 1, 3 and 5 days. The other detailed procedures were nearly similar to the procedures of the cell attachment test. The modified OD values on days 3 and 5 were normalized to the OD values on day 1 because the numbers of attached cells on the different materials were different on day 1.

The cell morphology on the samples was observed via confocal laser scanning microscopy (CLSM). After the cells were cultured on the samples for 5 days, the cells were gently washed three times with PBS, fixed with 4% paraformaldehyde for 15 min, permeabilized with 0.1% Triton X-100 in PBS for 10 min and stained with rhodamine phalloidin (5 U/mL, Biotium, Hayward, CA, USA) for 30 min. The filamentous actin of the cell cytoskeleton was visualized using a CLSM (TCS SP2, Leica, Heidelberg, Germany).

### Alkaline phosphatase (ALP) activity assay

The MC3T3-E1 cells were seeded on the composite samples at a density of 3 × 10^4^/cm^2^ in a six-well plate containing samples (Φ10 × 2 mm). After the cells were cultured for 24 hours, the culture medium was changed to an osteogenic inductive culture medium supplemented with 100 nM dexamethasone (Sigma-Aldrich), 50 μg/mL ascorbic acid (Sigma-Aldrich) and 10 mM β-glycerophosphate sodium (Sigma-Aldrich). After 7 and 14 days, ALP activity was determined by quantifying the amount of p-nitrophenol using a microplate test kit (Nanjing Jiancheng Bioengineering Institute, Nanjing, China), and the ODs were measured using a microplate reader at 405 nm. Furthermore, the total protein content was determined using the BCA protein assay kit (Pierce, Thermo, Rockford, Illinois, USA) according to the manufacturer’s provided. The ALP activity was finally normalized to the corresponding content of total protein.

### Statistical analysis

All data are presented as the means ± standard deviations, and all experiments were repeated three times. Statistical significance between different groups was analyzed using a two-way analysis of variance (ANOVA) test, and multiple comparisons were performed using the least significant difference (LSD) test. A p value < 0.05 was defined as significant, and a p value < 0.01 was defined as highly significant.

## Electronic supplementary material

Dataset 1
