# Supplementary material for: Influences of surface treatments with abrasive paper and sand-blasting on surface morphology, hydrophilicity, mineralization and osteoblasts behaviors of n-CS/PK composite
Source: Sci Rep. 2017 Apr 3;7:568. doi: 10.1038/s41598-017-00571-4 (PMC5428562; doi:10.1038/s41598-017-00571-4)
Supplement: Supplementary file 1 — Dataset 1 [file 41598_2017_571_MOESM1_ESM.doc]

**Influences of surface treatments with abrasive paper and sand-blasting on surface morphology, hydrophilicity, mineralization and osteoblasts behaviors of n-CS/PK composite**

Xiaoming Tang^1^, Kai Huang^2#^, Jian Dai^1^, Zhaoying Wu^3^, Liang Cai^3^, Lili Yang^4^, Jie Wei^3^, Hailang Sun^1*^

^1^Department of Orthopedics, Huai’an First People’s Hospital, Nanjing medical university, Huai’an 223001 Jiangsu Province, China

^2^Department of orthopedics, Shanghai Zhabei Central Hospital, Shanghai 200070, China

^3^Key Laboratory for Ultrafine Materials of Ministry of Education, East China University of Science and Technology, Shanghai 200237, PR China

^4^ Department of Orthopedic Surgery, Changzheng Hospital, Second Military Medical University, Shanghai 20003, China

# Kai Huang contributed equally to the article with the first author

*Address correspondence to: Hailang Sun ([sunhailang2016@sina.com](mailto:sunhailang2016@sina.com))

Tel.: +86-517-84907287; Fax: +86-517-84907287

**Supporting information**

Supplementary Table 1 The Ra values for the surface roughness of CSPKC, 12-CSPKC, 8-CSPKC, 4-CSPKC and sb-CSPKC.

| Samples | Ra (μm) |
| --- | --- |
| CSPKC | 1.58 |
| 12-CSPKC | 1.06 |
| 8-CSPKC | 1.13 |
| 4-CSPKC | 1.48 |
| sb-CSPKC | 3.82 |
